# Self-Report Measures of Procrastination Exhibit Inconsistent Concurrent Validity, Predictive Validity, and Psychometric Properties

**DOI:** 10.3389/fpsyg.2022.784471

**Published:** 2022-02-24

**Authors:** Lisa Vangsness, Nathaniel M. Voss, Noelle Maddox, Victoria Devereaux, Emma Martin

**Affiliations:** ^1^Department of Psychology, Wichita State University, Wichita, KS, United States; ^2^FMP Consulting, Arlington, VA, United States

**Keywords:** procrastination, pacing styles, psychometrics, predictive validity, concurrent validity, self-report measures

## Abstract

Procrastination is a chronic and widespread problem; however, emerging work raises questions regarding the strength of the relationship between self-reported procrastination and behavioral measures of task engagement. This study assessed the internal reliability, concurrent validity, predictive validity, and psychometric properties of 10 self-report procrastination assessments using responses collected from 242 students. Participants’ scores on each self-report instrument were compared to each other using correlations and cluster analysis. Lasso estimation was used to test the self-report scores’ ability to predict two behavioral measures of delay (days to study completion; pacing style). The self-report instruments exhibited strong internal reliability and moderate levels of concurrent validity. Some self-report measures were predictive of days to study completion. No self-report measures were predictive of deadline action pacing, the pacing style most commonly associated with procrastination. Many of the self-report measures of procrastination exhibited poor fit. These results suggest that researchers should exercise caution in selecting self-report measures and that further study is necessary to determine the factors that drive misalignment between self-reports and behavioral measures of delay.

## Introduction

Procrastination is a chronic and widespread problem, with some studies suggesting that one of every five adults engage in the behavior (Ferrari et al., 2007). Commonly, procrastination is identified through peoples’ responses to self-report instruments. While research suggests these self-report instruments are strongly correlated with one another (e.g., Ferrari et al., 2005; Svartdal and Steel, 2017), emerging work raises questions regarding the strength of the relationship between self-reported procrastination and behavioral measures of task engagement (Krause and Freund, 2014; Kim and Seo, 2015; Imhof et al., 2021) or pacing (Vangsness and Young, 2020; Voss and Vangsness, 2020). Relatedly, recent research (Hussey and Hughes, 2020) and policy (Sackett et al., 2018) underscored a need to assess the psychometric properties and predictive validity of commonly used self-report instruments. Ideally, such tests would control for possible sources of variability such as sample characteristics, administration method, or task type (for a summary see Kim and Seo, 2015). To our knowledge, such a test has not been conducted on self-report measures of procrastination.

This paper summarizes literature documenting the alignment among self-report measures of procrastination (i.e., concurrent validity) and between self-report and behavioral measures of procrastination and pacing (i.e., predictive validity). We review several possible statistical and psychometric explanations for discrepancies that exist and then explore these explanations by testing the internal reliability, concurrent validity, predictive validity, and psychometric properties of 10 self-report measures of procrastination. In our discussion, we review our findings and offer several additional explanations for the inconsistencies we observed in the psychometric properties and predictive validity of self-report measures of procrastination.

### Documented Relations Among Self-Reported Procrastination, Performance Measures, and Behavioral Delay

Historically, researchers have observed strong relationships among self-report measures of procrastination (e.g., Ferrari and Emmons, 1995; Specter and Ferrari, 2000; Sirois, 2007; Hen and Goroshit, 2018). This is an intuitive finding, given that many self-report measures of procrastination are created using items from other assessments and seek to assess the same underlying latent construct. For example, the Pure Procrastination Scale (PPS; Steel, 2010) includes items from Mann et al. (1997). Decisional Procrastination Scale, Lay’s (1986) General Procrastination Scale, and the Adult Inventory of Procrastination (AIP; McCown et al., 1989). The PPS correlates highly with these instruments, with participants’ scores on the PPS explaining at least 66% of the variability in their scores on the other instruments (Svartdal and Steel, 2017). Less intuitively, related measures share common method variance, which can inflate the strength of raw correlations between similar measures (Podsakoff et al., 2003).

In contrast, evidence for the predictive validity of self-report measures is mixed. Recent meta-analytic work suggests that self-report measures of procrastination demonstrate moderate predictive validity with measures of performance. Kim and Seo (2015) found that overall, self-report measures of procrastination explained only 2–11% of the variability in peoples’ performance (i.e., significant correlation coefficients ranged from −0.13 to −0.33), depending on which procrastination instrument was used by the researcher. The strength of this relationship also depended on the measure of performance used by the researcher. For example, GPA explained only 1.4% of the variability in peoples’ procrastination self-reports (*r* = –0.12), whereas individual assignment grades could account for 42% of this variance (*r* = –0.65). Interestingly, the strength of these relationships also depended on how the predictors were assessed. Self-report measures of procrastination shared a non-significant relationship with self-reported measures of performance (*r* = –0.08; 1% of variance explained) and were only moderately correlated with behavioral measures of performance (*r* = –0.15; 2% of variance explained). When procrastination and performance were both behaviorally assessed, the effect size was larger (*r* = –0.39; 15% of variance explained).

Relatedly, it is useful to consider the relationship between procrastination and pacing styles, “behavioral tendencies regarding the distribution of effort over time” (Gevers et al., 2015). Unlike procrastination, in which a person engages in a conscious and willful delay despite their knowledge of its negative outcomes (Klingsieck, 2013), pacing styles are defined more broadly and without negative connotation (Bloun and Janicik, 2002; Gevers et al., 2015). Endorsement of a deadline action pacing style—putting off task engagement until just before a deadline—is observed to correlate with self-reported procrastination (*r* = 0.55, or 30% of variance explained; Gevers et al., 2015). Although many researchers consider the deadline action pacing style as distinct from procrastination, self-report measures of procrastination frequently test predictive validity using behavioral measures of pacing style.

**FIGURE 1:**
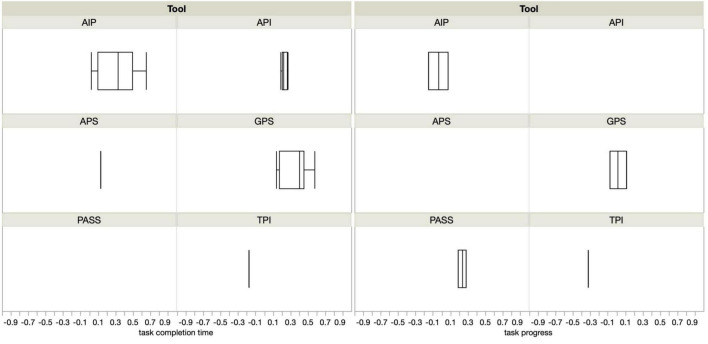
Distributions of correlation coefficients depict the strength of the relationship between task completion time (left), task progress (right), and self-report measures of procrastination.

While published literature provides support for the relationship between self-report measures of procrastination and behavioral measures of pacing style, the size of these effects are mixed. Most of the zero-order correlation coefficients identified in our table research ranged from *r* = 0.17 to *r* = 0.45 (3–20% of variance explained; see Figure 1). Some large effect sizes do exist and are illustrated by the whiskers in Figure 1, but they are inconsistent across samples: Ferrari (1992) found that the Adult Inventory of Procrastination (AIP) explained 42% of the variance in the number of days it took non-traditional students to return a folder; however, this zero-order correlation was less robust for the sample of traditional college students, explaining only 16% of variability in the same behavior. In this same study, the AIP explained at most 2% of the variance in the time it took students to complete their final exam. More recently, Zuber et al. (2021) found that the voluntary and observed delay subscales of the PPS shared a significant correlation with the number of days it took students to email their instructors a signed document (*r*’s = 0.41 and 0.47, respectively). Independently, these zero-order correlations explained 17% and 22% of the variability in students’ behavior; when regressed together, they explained 27% of the variance in behavior. Steel et al. (2018) also observed rather high correlations between the IPS and a behavioral measure of pacing: area under the curve created by plotting students’ cumulative assignment completion (*r* = 0.41, 17% of variance explained).

### Statistical and Psychometric Explanations for Discrepancies Among Measures of Procrastination

The examples of published zero-order correlation coefficients, discussed in the previous paragraphs, test the predictive and concurrent validity of self-report measures of procrastination. Although all of the reported coefficients were significant at an alpha of .05, statistical significance does not necessarily provide support for concurrent or predictive validity. To interpret the zero-order correlation coefficients, readers must first consider the researchers’ sample size, the number of statistical tests that were conducted, the psychometric properties of the scales that are being used, and the impact of nuisance variables on behavioral data.

#### Sample Size

Large samples have greater power to detect a statistically significant relationship between two predictors, even when that relationship is small. For example, Meehl (1990) described the results he obtained from a large-scale (*N* = 57,000) state survey database. Of the 990 analyses he conducted on this data, 92% were statistically significant. Meehl (1990) noted that these were not Type I errors, but rather stable—but small—relationships observed within the sample. That these relationships were statistically significant says more about researchers’ certainty in the presence of an effect than it does about whether the effect is large enough to be meaningful or generalize out-of-sample. It is possible that a similar issue affects the generalizability of procrastination studies with extremely large samples.

For this reason, many statisticians promote effect size over statistical significance (e.g., Royall, 1986; Tukey, 1991; Cohen, 1994; Kirk, 1996), with an emphasis on the “practical significance” (Royall, 1986; Kirk, 1996) of the effects. Relatedly, meta-analyses, such as Kim and Seo’s (2015) work, can allow researchers to identify stable relationships and determine how they change relative to study characteristics such as instrument and sample characteristics (Kirk, 1996). This may help explain why overall, self-report measures of procrastination explained only 1% of the variance in peoples’ self-report performance and 2% of the variance in peoples’ behavioral measures of performance, but that individual measures (e.g., specific assignment grades) fared much better (Kim and Seo, 2015).

#### Number of Statistical Tests

A separate explanation for the inconsistent relationship between self-report and behavioral measures has to do with inflated Type I error rates associated with many pairwise comparisons. A typical psychology publication reports a Type I error rate of .05. Functionally, this threshold requires the researchers—and the reader—to assume the risk that a single significant relationship has a 5% chance of being a false discovery. This error rate is unique to each statistical test; therefore, in papers that report multiple tests (e.g., all possible pairwise comparisons; multiple correlation tables; etc.), the Type 1 error rate is equal to 1−(1−α)*^c^*, where c represents the number of tests conducted (Keppel and Wickens, 2004, p. 112). For example, one of the studies reported in this paper contains 14 statistical tests, of which four were statistically significant. This set of analyses ran at least a 51% chance of making a false discovery. Therefore, it is possible that some of the significant relationships that emerge in studies that conduct multiple pairwise comparisons will not generalize—or will appear as a smaller effect—in a different sample.

#### Psychometric Properties

Another important consideration for assessing the relationship between self-report measures of procrastination and behavioral outcomes concerns the nature of self-report measures themselves. As noted above, self-report measures of procrastination are not typically strong predictors of behavior (but see Zuber et al., 2021). Poor psychometric properties are one potential explanation for why self-report measures may not adequately predict behavior. For a measure to be valid, it must possess acceptable psychometric properties such as high reliability, good model-data fit, and measurement invariance (MI; e.g., Hussey and Hughes, 2020). It must also be related to theoretically relevant constructs (i.e., possess concurrent validity) and unrelated to theoretically irrelevant constructs (i.e., possess discriminant validity). When a measure is lacking any of these properties, it lacks sufficient evidence for construct validity and does not sufficiently measure what it is supposed to be measuring. If a measure is not properly assessing procrastination, it is unlikely to predict procrastination behavior. Of course, it is still possible for self-report measures with poor psychometric properties to be significantly related to other variables. In such cases, these relations are meaningless since the measures themselves lack evidence of construct validity.

#### Impact of Nuisance Variables on Behavioral Data

A final explanation for the small effects observed in the literature is the influence of nuisance variables on behavioral measures of delay and performance. Previous empirical (e.g., Epstein, 1979; Abelson, 1985) and philosophical (Epstein, 1983) works note that behavioral measures are influenced by a variety of uncontrolled factors (i.e., nuisance variables such as competing tasks or fatigue) that increase error variance and can obscure the true relationship between self-report and behavioral measures. In such cases, repeated-measures approaches to data analysis—such as aggregation (Epstein, 1983) or multi-level modeling (Gelman and Hill, 2006)—statistically control for nuisance variance and yield estimates that are more reflective of the true relationship between self-report and behavioral measures. This is especially true for traits, which influence behavior but can be overshadowed by context-specific factors (Mischel, 1977). If nuisance variables obscure the relationship between self-report measures of procrastination and behavioral measures of delay or performance, aggregated measures (e.g., GPA, pacing style, course grade) should hold stronger relationships with self-report measures than do disaggregated measures (e.g., assignment grade). Interestingly, this appears to be the opposite relation as was observed among measures of performance in Kim and Seo’s (2015) meta-analysis. Nevertheless, it is worthwhile to consider whether single index measures of procrastination (e.g., completion date) perform more poorly than aggregate measures (e.g., behavioral measures of pacing style).

### The Current Study

Understanding the relationship between self-report measures of procrastination and behavioral outcomes requires more than simply examining the zero-order correlations between these two types of measures. It also requires a holistic consideration of sample size, the number of tests conducted, and the psychometric properties/construct validity of the measures that are employed. Therefore, we chose to assess the convergent and predictive validity of 10 self-report measures of procrastination using cluster analysis (Gore, 2000) and Lasso estimation (Tibshirani, 1996), two statistical approaches that control for the family-wise errors inherent in repeated analysis and the multicollinearity present among related measures. The study was conducted over the course of an entire semester and involved a task with a distant deadline, providing ample opportunity to measure procrastination and students’ pacing styles. We also examined the psychometric properties of 10 self-report procrastination measures via confirmatory factor analysis (CFA).

## Materials and Methods

### Participants

Research participants were psychology students currently enrolled at Wichita State University. All research procedures were approved by the Institutional Review Board at Wichita State University. Qualtrics was used to obtain participants’ consent and to administer an online battery of self-report instruments to 242 students; seven did not click through to the end of the survey and were excluded from analyses. The remaining 235 participants (μ_*age*_ = 22, *SD*_age_ = 7.14) completed all 10 survey instruments in exchange for four research credits. Demographic information about these participants is available in Table 1.

**TABLE 1:** Sample demographic information.

Demographic characteristic	*N*
**Sex**	
Female	165
Male	63
Other	2
Prefer not to say	5
**Race**	
White	155
Black	22
Asian	30
Hispanic/Latinx	24
Native Hawaiian/Pacific Islander	1
American Indian	0
Prefer not to say	3

### Procedure

Participants completed an online survey comprised of 10 well-known self-report measures of procrastination (for details, see section “Self-Report Measures”). Five directed response (e.g., Please select “Agree” when responding to this item.) and five bogus items (e.g., I do not understand a word of English.) were evenly spaced throughout the battery to ensure that participants were paying attention (Meade and Craig, 2012). Students answered five, fixed-order demographics questions before completing the procrastination surveys—and the items within them—in a randomized order. A 5 (*strongly agree*) to 1 (*strongly disagree*) scale was adopted for all measures except the PASS, which employed a 5 (*always*) to 1 (*never*) scale; higher scores indicated greater levels of procrastination. Surveys were evenly administered over the course of the 16-week semester to ensure that the study would be completed by students exhibiting a variety of pacing styles. The survey took around 30 min to complete and could be returned to at any time for up to a week. A full copy of the survey and its randomization information can be found at the project’s OSF page: https://osf.io/r7wcx/.

#### Self-Report Measures

Self-report measures of procrastination were selected with an eye toward utility. The instruments that follow are widely used by researchers studying procrastination, and all have undergone some degree of validation.

##### Metacognitive Beliefs About Procrastination

The MBP is a 14-item survey that requires people to endorse statements about the emotions and thoughts that drive procrastination (e.g., “Procrastination stops me from making poor decisions when I am feeling anxious”; Fernie et al., 2009). Participants’ responses to these items were averaged together to create a composite score that ranged from 1 to 5, where higher scores indicated more positive beliefs about procrastination. This instrument was initially validated through exploratory and CFA, as well as internal consistency reliability (Fernie et al., 2009).

##### Academic Functional Procrastination

The AFP is a 9-item survey that invites respondents to endorse statements related to procrastination within an academic context (e.g., “I have procrastinated on my lessons in some cases in order to be motivated for them”; Kandemir and Palanci, 2014). Participants’ responses to these items were averaged together to create a composite score that ranged from 1 to 5, where higher scores indicated a stronger endorsement of statements related to a strategic use of academic procrastination. This instrument was originally validated through exploratory factor analysis, CFA, and internal consistency reliability (Kandemir and Palanci, 2014).

##### Lay’s General Procrastination Scale

The 16-item GPS allows participants to endorse general statements about procrastination (e.g., “I usually make decisions as soon as possible”; Lay, 1986). Participants’ responses to these items were averaged together to create a composite score that ranged from 1 to 5, where higher scores indicated a stronger endorsement of statements related to procrastination. This instrument’s initial validation involved tests of predictive, concurrent, and divergent validity (Lay, 1986). Subsequent factor analysis and principal components analysis have been conducted by Vesterveldt (2000), Svartdal and Steel (2017), and Klein et al. (2019).

##### Adult Inventory of Procrastination

The 10-item AIP requires respondents to rate the degree to which they endorse general statements about procrastination (e.g., “My friends and family think I often wait until the last minute”; McCown et al., 1989). Participants’ responses to these items were averaged together to create a composite score that ranged from 1 to 5, where higher scores indicated a stronger endorsement of statements related to procrastination. This instrument was initially validated through principal components analysis (McCown et al., 1989). Subsequent factor analysis and principal components analysis have been conducted by Vesterveldt (2000) and Svartdal and Steel (2017).

##### Active Procrastination Scale

The APS invites participants to indicate the degree to which they use procrastination as a motivational tool (e.g., “I intentionally put off work to maximize my motivation”; Choi and Moran, 2009). Participants’ responses to these items were averaged together to create a composite score that ranged from 1 to 5, where higher scores indicated a stronger endorsement of statements related to active procrastination. This instrument was originally validated through assessments of concurrent and divergent validity, exploratory and CFA, internal consistency reliability, and predictive validity (Choi and Moran, 2009). Subsequent validation has been conducted by Pinxten et al. (2019).

##### Unintentional Procrastination Scale

The UPS is a 7-item assessment that requires participants to endorse general statements about procrastination (e.g., “I often seem to start things and don’t seem to finish them off”; Fernie et al., 2017). Participants’ responses to these items were averaged together to create a composite score that ranged from 1 to 5, where higher scores indicated a stronger endorsement of statements related to procrastination. This instrument was initially validated through principal components analysis, concurrent and divergent validity, exploratory and CFA, and internal consistency reliability (Fernie et al., 2017).

##### Procrastination Assessment Scale for Students

The PASS is a 12-item assessment that allows people to rate the degree to which they procrastinate on school-related tasks (e.g., “To what degree is procrastination on *attendance tasks* a problem for you?”; emphasis original; Solomon and Rothblum, 1984). Participants’ responses to these items were averaged together to create a composite score that ranged from 1 to 5, where higher scores indicated a stronger endorsement of statements related to procrastination for academic tasks. This instrument has been intentionally validated through predictive validity, exploratory factor analysis, and concurrent validity (Solomon and Rothblum, 1984); subsequent factor analyses have been conducted by Yockey and Kralowec (2015).

##### Irrational Procrastination Scale

The IPS is a 9-item scale that asks participants to endorse statements related to the irrationality of procrastination (e.g., “At the end of the day, I know I could have spent the time better.”; Steel, 2010). Participants’ responses to these items were averaged together to create a composite score that ranged from 1 to 5, where higher scores indicated a stronger endorsement of statements related to procrastination. This instrument has been intentionally validated through exploratory factor analysis, CFA, and internal consistency reliability (Steel, 2010).

##### Pure Procrastination Scale

The PPS is a 12-item scale that requires participants to endorse general statements about procrastination (e.g., “I am not very good at meeting deadlines”; Steel, 2010). Participants’ responses to these items were averaged together to create a composite score that ranged from 1 to 5, where higher scores indicated a stronger endorsement of statements related to procrastination. This instrument was initially validated through exploratory factor analysis, CFA, and internal consistency reliability (Steel, 2010). Subsequent factor analysis has been conducted by Svartdal and Steel (2017).

##### Tuckman’s Procrastination Scale

The TPS is a 16-item instrument that invites participants to endorse general statements related to procrastination (e.g., “I get stuck in neutral even though I know how important it is to get started; Tuckman, 1991). Participants’ responses to these items were averaged together to create a composite score that ranged from 1 to 5, where higher scores indicated a stronger endorsement of statements related to procrastination. This instrument was initially validated through exploratory factor analysis, CFA, internal consistency reliability, and predictive validity (Tuckman, 1991).

#### Behavioral Measures of Delay

In addition to providing self-reports, we also employed two behavioral measures of delay to test the predictive validity of the self-report measures of procrastination. These measures were derived from the dates of students’ research appointments recorded in the Sona Systems database (Sona Systems, 2021), our institution’s experiment management system. Research participation requirements are shared in syllabi, worth course credit, and have deadlines. The students in our sample all needed to complete 16 credits before the end of the semester. These students were aware that failing to complete their research credits would be disadvantageous—it would negatively impact their grade—and were reminded of this fact several times throughout the semester. Research appointments were available throughout the semester. Therefore, delaying the completion of a single research credit by a few weeks (especially early in the semester) would not place students in danger of failing the assignment. However, a pattern of delay exhibited across the course of the semester would, as research appointments are a limited resource. These circumstances gave rise to “weak” situations (Mischel, 1977) in which individual differences in pacing style were expected to be especially pronounced (Gevers et al., 2015). Thus, these measures represented a meaningful way to test predictive validity of these self-report instruments (Vangsness and Young, 2020).

##### Days to Study Completion

Days to study completion was assessed by computing the number of days into the semester on which a student completed our research study, according to the registrar’s calendar and the records from the SONA system. Each semester was 16 weeks (112 days) long. Researchers have used similar measures of delay to demonstrate the predictive validity of self-report measures (e.g., Solomon and Rothblum, 1984; Lay, 1986; Ferrari, 1992; Reuben et al., 2015) or to identify procrastination behavior among participants (e.g., Steel et al., 2001).

##### Pacing Styles

Pacing styles were assessed by treating students’ research participation records as a distribution that could be described by three characteristics: task initiation (day of first research credit completion), central tendency (average completion day), and spread (completion distribution; *SD*). We subjected these three measures to a latent profile analysis (LPA), using the procedure outlined in Vangsness and Young’s (2020) methodological paper. Students’ profile membership was then used to create a binary outcome variable where students who exhibited a deadline action pacing style were assigned a 1 and all other students (i.e., precrastinators/early action pacing and steady working) were assigned a 0.

### Statistical Approach

Psychometric and predictive validations were conducted in R (R Core Team, 2020) as two-tailed tests with an alpha of 0.05, while the cluster analysis was conducted with JMP Pro 15. All procedures are freely available through the project’s OSF page, and the analyses can be reproduced using the provided data file, which has been anonymized and stripped of identifying information to preserve students’ confidentiality.

#### Selection of Sample Size

*Post-hoc* power analyses were conducted for all analyses. The study sample provided between 58 and 88% power to detect the observed effects. Code and graphical illustrations of the power analyses are available on the project’s OSF page.

#### Psychometric Analyses

The quality of the self-report measures of procrastination was assessed via a series of CFA models and MI analyses (Vandenberg and Lance, 2000). The CFA models were estimated to align with the factor structures proposed by the authors of these scales. To evaluate the fit of these models, we relied on standard thresholds for several model fit indices (e.g., CFI ≥ 0.95, RMSEA ≤ 0.08; Hu and Bentler, 1999). We conducted MI analyses on the scales by using pacing style as the grouping variable (see Voss and Vangsness, 2020): those who employed a deadline action pacing style and those who did not (i.e., engaged in early action or steady work pacing). We employed a CFA approach by estimating a series of increasingly constrained models where the factor structures (configural invariance), factor loadings (metric invariance) and item intercepts (scalar invariance) were constrained to be equal across groups. When comparing models, we relied on both Δχ^2^-values and ΔCFI values since χ^2^ is overly sensitive to sample size (Brown, 2006). We considered models with a significant Δχ^2^-value and ΔCFI > 0.01 as representing a substantive difference between models (Vandenberg and Lance, 2000).

#### Cluster Analysis

K-means cluster analysis (Gore, 2000; Wu, 2012) was used to determine the degree to which participants’ self-report composite scores aligned with one another. We employed the standard measure of similarity (Euclidean distance) and used Cubic Clustering Criteria (CCC) values to select an appropriate clustering solution. Because participants’ responses to the survey instruments were collected and averaged using a 5-point scale, further standardization of the data was not necessary.

#### Lasso Estimation and Regressions

We assessed the relationship between the self-report and behavioral measures of delay with lasso estimation. The lasso modeling approach incorporates a penalty against individual parameter estimates, encouraging sparser models. This approach is particularly useful when there is low-to-moderate multicollinearity among the predictors of a dataset (Tibshirani, 1996; Schreiber-Gregory and Jackson, 2017), as was the case in this study. The estimation penalty (i.e., lambda) was selected using the cross-validation function provided by the glmnet package in R. This cross-validation function runs a 10-fold cross-validation on the data using 100 possible values of lambda. The selected value is the one that minimizes the mean cross-validated error. We planned to follow up on the relationships identified by the lasso with logistic and Poisson regressions, which take into account the unusual error distribution exhibited by binomial (i.e., deadline action vs. other pacing styles) and count variables (i.e., days to study completion). These analyses provided easier-to-interpret parameter estimates, standard errors, and significance values.

## Results

### Careless Responding

Participant responses to the attention check items in our survey indicated that our participants were well-attentive: 160 Students answered all these questions correctly, and 60 students missed only one of the questions. Therefore, all data were retained for subsequent analyses.

### Internal Reliability

Cronbach’s (1951) alpha was used to assess the internal reliability of self-report measures of procrastination.^1^ In general, all of the procrastination measures exceeded the recommended threshold of 0.70 (Lance et al., 2006) and bore similarity to those reported in past studies (see Table 2). In general, participants’ responses were well-aligned within a single instrument.

**TABLE 2:** Current and historic measures of reliability for self-report procrastination assessments.

Self-report measure	α_current_	α_historic_
Metacognitive Beliefs about Procrastination (MBP)		
Positive Beliefs Subscale	0.74	0.81
Negative Beliefs Subscale	0.80	0.85
Academic Functional Procrastination (AFP)	0.79	–
Lay’s General Procrastination Scale (GPS)	0.87	0.82
Adult Inventory of Procrastination (AIP)	0.83	0.76
Active Procrastination Scale (APS)	0.76	0.80
Unintentional Procrastination Scale (UPS)	0.83	–
Procrastination Assessment Scale for Students (PASS)	0.90	0.85
Irrational Procrastination Scale (IPS)	0.85	0.91
Pure Procrastination Scale (PPS)	0.90	0.86
Tuckman’s Procrastination Scale (TPS)	0.93	0.90

*Cronbach’s alpha was not reported in the original publications of the AFP, UPS, and PASS; Solomon and Rothblum (1984) did not assess the internal reliability of this measure in their original publication; subsequent alphas range from 0.66 to 0.85.*

### Concurrent Validity of Self-Report Measures

**FIGURE 2:**
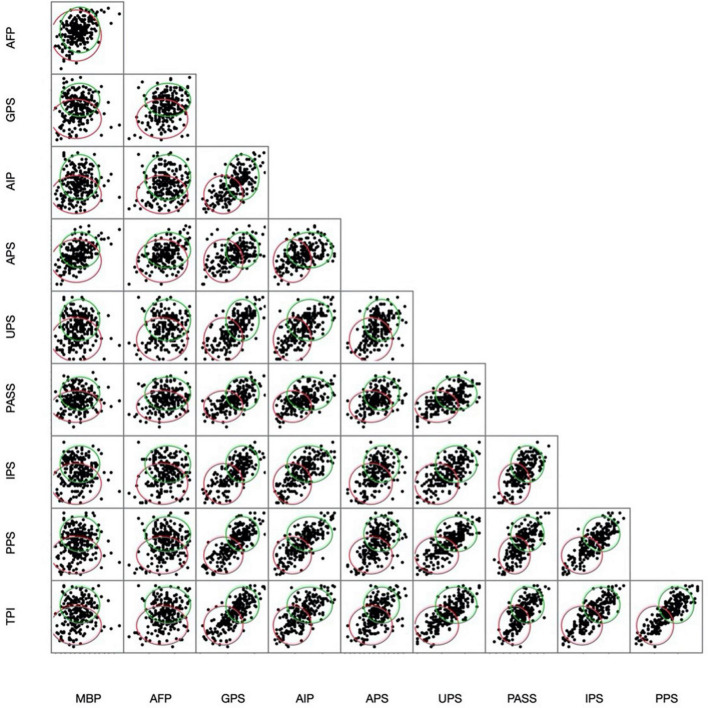
Respondents’ self-reports were best represented by a two-cluster solution that clearly delineated procrastinators (green; upper ellipse) from non-procrastinators (red; lower ellipse).

CCC values indicated a two-cluster solution was most appropriate for the data (see Figure 2). Cluster means (see Table 3) indicated that the two clusters could be characterized as follows:

**TABLE 3:** Cluster means illustrate the degree to which individual self-report measures of procrastination differentiated procrastinators from non-procrastinators.

Predictor	Procrastinators	Non-procrastinators	△
Pure Procrastination Scale (PPS)	3.51	2.18	1.33
Tuckman’s Procrastination Scale (TPS)	3.59	2.32	1.27
Unintentional Procrastination Scale (UPS)	3.57	2.34	1.23
Irrational Procrastination Scale (IPS)	3.70	2.55	1.15
Lay’s General Procrastination Scale (GPS)	3.45	2.40	1.05
Adult Inventory of Procrastination (AIP)	2.91	1.96	0.95
Procrastination Assessment Scale for Students (PASS)	3.43	2.49	0.94
Active Procrastination Scale (APS)	3.00	2.44	0.56
Academic Functional Procrastination (AFP)	3.72	3.39	0.33
Metacognitive Beliefs about Procrastination (MBP)	2.62	2.42	0.20

1. Procrastinators (*n* = 94): scored highly on forward-coded measures, excluding the PASS, which was reverse-coded.
2. Non-Procrastinators (*n* = 123): scored low on forward-coded measures, excluding the PASS.

Although these clusters were consistent with researchers’ current understanding of procrastination, differentiation was driven by some self-report measures more than others. This phenomenon is illustrated in Figure 2, which contains density ellipses that encircle 90% of the students included in each cluster. These ellipses are overlaid on a correlation matrix. The separation of the ellipses corresponds with the degree to which each set of assessments contributes to identifying procrastination among the members of the sample; in cases where instruments are highly correlated, the data and ellipses are spread along the diagonal. Cluster means revealed that classifications were predominantly driven by participants’ scores on the PPS, TPS, UPS, IPS, AIP, and GPS. This is illustrated by the clear separation in cluster ellipses across these panels in Figure 2. These measures were also highly correlated with one another (see Table 4), a relationship that is reflected in the diagonal separation of cluster ellipses for these measures. Therefore, the self-report measures largely exhibited concurrent validity with one another.

**TABLE 4:** Correlations between individual survey instruments and continuous behavioral measures.

	MBP	AFP	GPS	AIP	APS	UPS	PASS	IPS	PPS	TPS
MBP										
AFP	**0.47**									
GPS	0.15	**0.25**								
AIP	0.14	0.10	**0.66**							
APS	**0.55**	**0.46**	**0.49**	**0.44**						
UPS	0.07	0.13	**0.70**	**0.67**	**0.45**					
PASS	0.14	0.20	**0.69**	**0.59**	**0.48**	**0.63**				
IPS	0.09	0.14	**0.75**	**0.74**	**0.47**	**0.68**	**0.70**			
PPS	0.12	0.15	**0.78**	**0.76**	**0.49**	**0.79**	**0.71**	**0.82**		
TPS	0.15	0.19	**0.77**	**0.70**	**0.53**	**0.79**	**0.75**	**0.82**	**0.86**	
DSS	0.19	–0.02	0.13	0.06	0.15	0.09	0.21	0.18	0.16	0.19

*DSS = Days Since Start (behavioral delay). Values in bold are statistically significant at the 0.001 level (0.05 Bonferroni-corrected for multiple pairwise comparisons).*

### Pacing Styles

**FIGURE 3:**
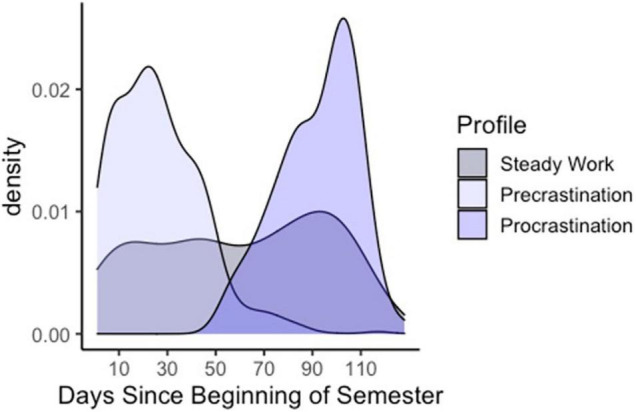
Students’ research credit completion strategies were identified as precrastination, steady work, and procrastination.

The pacing styles identified by our LPA mirrored those found previously (see Figure 3). Specifically, one latent profile (“precrastination”) represented students who engaged in an early action pacing style by starting their research credits early and quickly completing them at the beginning of the semester. Another profile (“steady work”) represented students who engaged in a steady work pacing style by starting their research credits early and methodically participating in research until the end of the semester. The last profile (“procrastination”) represented students who exhibited a deadline action pacing style by starting their research credits late in the semester and quickly completing them before the deadline. These patterns were similar to those found in previous work despite in-person classes being canceled due to the COVID-19 pandemic. This is likely to due to the increased number of online studies available at the first author’s institution. Interestingly, the number of people engaging in procrastination-like task completion strategies (*n* = 57) was lower than the number of procrastinators identified by cluster analysis, a classification strategy that relied solely on self-report measures.

### Confirmatory Factor Analysis

As seen in Table 5, the CFA (estimated via maximum likelihood) fit of these models (labeled “*Baseline Fit*”) was generally unacceptable apart from a few scales (e.g., UPS, IPS, and TPS). The psychometric issues that we identified with the scales were inconsistent and included mis-specified factor structures, item cross-loadings, and highly collinear item residuals. In many ways, these results are not surprising given that similar issues have been observed for many of these scales within the previous literature (e.g., PASS, Yockey and Kralowec, 2015; GPS, Klein et al., 2019; APS Pinxten et al., 2019; AIP, Svartdal and Steel, 2017; PPS, Svartdal and Steel, 2017).

**TABLE 5:** Summary of the confirmatory factor analysis (CFA) and measurement invariance (MI) models for all self-report procrastination scales.

	Model fit indices				
Variable	χ^2^	AIC	CFI	RMSEA	Δχ^2^
**MBPS**					
*Baseline fit*	235.96**	8,130	0.80	0.10	–
Configural invariance	354.76**	8,201	0.76	0.11	–
Metric invariance	378.56**	8,200	0.75	0.11	23.79*
Scalar invariance	383.81**	8,182	0.76	0.11	5.25
**AFPS**					
*Baseline fit*	81.99**	5,157	0.86	0.10	–
Configural invariance	113.65**	5,205	0.85	0.11	–
Metric invariance	120.01**	5,195	0.86	0.10	6.35
Scalar invariance	121.94**	5,181	0.87	0.09	1.93
**LGPS**					
*Baseline fit*	235.48**	9,102	0.84	0.09	–
Configural invariance	358.01**	9,185	0.81	0.10	–
Metric invariance	368.62**	9,168	0.82	0.09	10.61
Scalar invariance	381.89**	9,153	0.82	0.09	13.28
**AIP**					
*Baseline fit*	154.49**	5,831	0.79	0.13	–
Configural invariance	203.17**	5,883	0.77	0.14	–
Metric invariance	208.10**	5,870	0.78	0.13	4.93
Scalar invariance	217.38**	5,861	0.78	0.12	9.28
**APS**					
*Baseline fit*	270.57**	9,551	0.83	0.09	–
Configural invariance	384.38**	9,650	0.82	0.10	–
Metric invariance	391.06**	9,633	0.82	0.09	6.68
Scalar invariance	398.84**	9,616	0.83	0.09	7.78
**UPS**					
*Baseline fit*	37.40**	4,253	0.94	0.09	–
Configural invariance	56.24**	4,293	0.93	0.10	–
Metric invariance	62.37**	4,287	0.93	0.09	6.13
Scalar invariance	67.06**	4,279	0.94	0.08	4.69
**IPS**					
*Baseline fit*	79.92**	5,087	0.91	0.10	–
Configural invariance	101.23**	5,123	0.92	0.09	–
Metric invariance	109.36**	5,115	0.92	0.09	8.13
Scalar invariance	124.87**	5,115	0.91	0.09	15.51
**PPS**					
*Baseline fit*	177.45**	6,993	0.89	0.11	–
Configural invariance	232.75**	7,051	0.89	0.11	–
Metric invariance	251.24**	7,051	0.88	0.11	18.49*
Scalar Invariance	260.10**	7,042	0.88	0.11	8.85
**TPS**					
*Baseline fit*	194.52**	9,044	0.94	0.07	–
Configural invariance	296.24**	9,114	0.94	0.06	–
Metric invariance	308.57**	9,096	0.94	0.06	12.33
Scalar Invariance	328.15**	9,086	0.94	0.06	19.59

*p < 0.05, **p < 0.01. Baseline fit refers to the fit across all groups. The grouping variable for the measurement invariance analysis consisted of the procrastinators and non-procrastinators (precrastinators and steady work groups) identified by the latent profile analysis. We were unable to estimate an appropriate model for the PASS.

### Measurement Invariance

Pacing style served as the grouping variable for the MI analyses. As seen in Table 5, a significant Δχ^2^-value was observed for two models (Metric invariance for the MBPS and the PPS). However, the ΔCFI for these models was not greater than 0.01, so it is unlikely that their fit deteriorated enough to represent a violation of MI. Thus, it appears that the psychometric properties of these self-report procrastination scales are largely equivalent regardless of one’s task completion strategy. Put another way, these findings suggest that the self-report procrastination scales function similarly (e.g., are interpreted in a similar manner) for people whose task completion strategies mirror those of procrastination and non-procrastination. Some caution is warranted in interpreting these results, however, since these groups were somewhat smaller than what is conservatively recommended for MI testing. More importantly, the baseline fit for most of the self-report scales was rather poor. Thus, aside from the few good fitting models (see Table 6), the MI results are unfortunately somewhat tenuous for many of the scales that we assessed.

**TABLE 6:** Summary of the results of psychometric analyses and tests of concurrent and predictive validity.

Instrument	Internal consistency	Measurement invariance	Acceptable model fit	Behavioral delay	Task completion strategy
MBP	*Y*	*Y*	*N*	*Y*	*N*
AFP	*Y*	*Y*	*N*	Y*	*N*
GPS	*Y*	*Y*	*N*	*N*	*N*
AIP	*Y*	*Y*	*N*	Y*	*N*
APS	*Y*	*Y*	*N*	*N*	*N*
UPS	*Y*	*Y*	*Y*	Y*	*N*
PASS	*Y*	*Y*	*N*	*Y*	*N*
IPS	*Y*	*Y*	*Y*	*Y*	*N*
PPS	*Y*	*Y*	*N*	*N*	*N*
TPS	*Y*	*Y*	*Y*	*Y*	*N*

*Asterisks denote weak predictive effects. Given the poor fit of many of the self-report measures, the results of the measurement invariance analyses for the poor-fitting models should be interpreted with caution.*

### Predictive Validity of Self-Report Measures

#### Pacing Styles

The lasso dropped all of the predictors (i.e., shrunk their regression weights to 0), suggesting that self-report measures of procrastination are not predictive of this behavioral measure of delay.

#### Days to Study Completion

**FIGURE 4:**
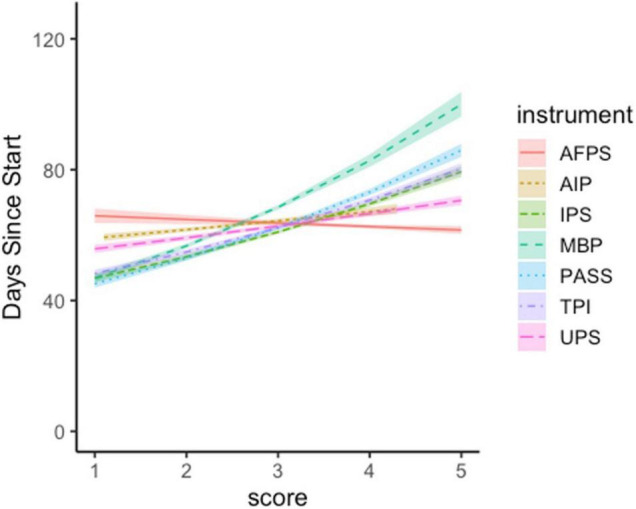
Respondents’ scores on many of the self-report measures of procrastination predicted the amount of time that elapsed before they completed the research study. The strength of this relationship differs across instruments. Error bars represent ±1 *SE*.

The lasso dropped only three self-report measures of procrastination: the GPS, the APS, and the PPS. Subsequent Poisson regressions indicated that days to study completion could be predicted by participants’ self-reports on the MBP (*B* = 0.19, *SE* = 0.02, *z* = 12.28, *p* < 0.001), the AIP (*B* = 0.04, *SE* = 0.01, *z* = 3.62, *p* < 0.001), the UPS (*B* = 0.06, *SE* = 0.01, *z* = 5.90, *p* < 0.001), the PASS (*B* = 0.16, *SE* = 0.01, *z* = 13.71, *p* < 0.001), the IPS (*B* = 0.13, *SE* = 0.01, *z* = 11.83, *p* < 0.001), and the TPS (*B* = 0.12, *SE* = 0.01, *z* = 12.24, *p* < 0.001). Students’ self-report responses to each of these measures shared a positive relationship with task-specific procrastination; however, the instruments did not exhibit equally strong effects, as evidenced by the slopes in Figure 4. The strongest predictor of task-specific delay was the MBP. Students who endorsed the items on this instrument most strongly completed the research study 53 days later than those who endorsed these items the least (100 and 47 days, respectively). The next strongest measure was the PASS, for which the highest- and lowest-scoring students differed by 41 days (86 and 45 days, respectively). Those who reported the highest levels of procrastination on the AIP completed the study 9 days later than those who reported the lowest levels of procrastination (68 and 59, respectively). The IPS and TPS were similarly predictive, with the highest scoring students on each instrument completing the study 32 days ahead of the lowest scoring students (79 and 47, respectively; 80 and 48, respectively). The least predictive instruments were the UPS and the AFPS, with the highest scoring students completing the study on average 15 and 4 days ahead of their lowest-scoring peers (56 and 71; 62 and 66, respectively). Poisson regression suggested that the relationship between the AFPS and the day on which participants completed the research study was weak and unlikely to generalize out-of-sample (*B* = –0.02, *SE* = 0.01, *z* = –1.36, *p* = 0.17).

## Discussion

Our results found that some self-report measures of procrastination exhibited better psychometric properties and displayed stronger predictive validity than did others. While all of the instruments exhibited good internal reliability, concurrent validity, and displayed MI, only the UPS, IPS, and TPS had consistent factor structures. We also discovered that the self-report measures predicted behavioral delay to varying degrees. Some of the measures we compared (i.e., MBP, PASS, TPS, IPS) were stronger predictors of behavioral delay than were others (i.e., UPS, AIP, AFP). A subset of measures (i.e., GPS, APS, and PPS) did not predict our behavioral measures of delay at all. Additionally, none of the self-report measures predicted engagement in a deadline action pacing style throughout the semester. This is especially notable, given that the deadline action pacing style is expected to be positively related to self-reported procrastination, especially when a task’s structure affords people great freedom to choose when and how they complete it (Gevers et al., 2006). While the poor predictive power of self-report measures is sometimes attributed to the presence of nuisance variables (e.g., Mischel, 1977; Epstein, 1979, 1983; Funder, 2012), our results were not consistent with this perspective. Therefore, we turn to alternative explanations for our observed relations.

Although the results can be partially explained by poor psychometric properties (e.g., the GPS), this is not true of all instruments. Therefore, it is likely that other factors contribute to the discrepancies we observed. Students may lose self-awareness of their task completion strategies when they are distributed over time, leaving their self-reports poorly predictive of their pacing style. Alternatively, pacing style and procrastination may be entirely unrelated constructs. Finally, some of these results may be explained by the poor psychometric properties of the instruments.

### Students May Lack Awareness of Their Task Completion Strategies

Procrastination has been described as an irrational behavior that occurs when a person fails to complete tasks that they know are in their best interests (Silver and Sabini, 1981; Steel, 2007, 2010). Some research suggests that this irrationality becomes clearer in hindsight (Kahneman and Tversky, 1981; Denes-Raj and Epstein, 1994). This form of bias is widely referred to as secondary hindsight bias (Kelman et al., 1998; Fischhoff, 2003), whereby a person revises their initial estimate of an event’s occurrence to align with an observed outcome. In the case of procrastination, a student may initially underestimate the likelihood that they will procrastinate on a term paper at the beginning of the semester. When asked about their academic habits at the end of the semester, the student upwards adjusts their estimate and acknowledges themselves as a procrastinator. What’s more, the student wrongfully believes that they would have described themselves as a procrastinator at the beginning of the semester. Although this belief contradicts the student’s earlier report, it results from updating estimates and certainty in light of new information (Kelman et al., 1998; Malatincová, 2015). Future research could test for secondary hindsight bias by administering questionnaires at task assignment and follow-up.

This perspective on irrationality aligns with definitions provided by the field of decision science wherein irrationality is something that can unfold—knowingly or unknowingly—as a byproduct of environmental, cognitive/perceptual, and personal constraints (Brunswik, 1943). Information about the rewards and consequences of task completion is not always available (c.f., Stevens, 1957), nor are the probabilities of success always known (Hau et al., 2010). For example, at the beginning of the semester, students are often unaware of how a final paper assignment will impact their grade. As the student completes and receives grades on assignments, they gain awareness of the paper’s impact and their abilities in the class. A student who is failing would have to expend effort to pass the class with a C+ paper; a student who has made straight A’s all semester may still receive an A even if they fail to turn the paper in. Indeed, factors such as these can impact the accuracy of a person’s self-referential judgments (e.g., Funder, 2012). For some students, the effort (Mitchell, 2017) and delayed rewards associated with the final paper may seem less attractive than the more immediate benefits of replying to an email or enjoying a game of Dungeons and Dragons with friends (Ainslie, 1975). Individual differences such as these are of particular importance in task completion decisions, which require a person to integrate and assign value to information across several domains (Kurzban et al., 2013) and windows of time (Grund and Fries, 2018).

This perspective could explain the divergence in our results whereby some self-report measures of procrastination could predict days to study completion, but none of the self-report measures predicted students’ pacing styles. Students who participated in our research study at the beginning of the semester might have misestimated the degree to which they would procrastinate at the end of the semester. This would produce an alignment between their self-report scores and their completion date and a divergence between self-report scores and their pacing style. There is some evidence for this perspective in studies that involve self-reports of dilatory behavior (e.g., Rothblum et al., 1986; Lay and Burns, 1991; Neo and Skoric, 2009; Lubbers et al., 2010; Malatincová, 2015; Liborius et al., 2019); however, the results of this study suggest that it is worthwhile to explore this effect further using objective single-index measures of delay.

Relatedly, it is useful to note that perceptions of procrastination may depend on the reference task (Vangsness and Young, 2020). For example, a student may have delayed their research participation so that they could study for their midterm exams. This student may self-report procrastination when thinking about their research participation, but not when thinking about their exam preparation. This line of reasoning could also explain the lack of relationship between self-reported procrastination and pacing style.

### Pacing Style and Procrastination May Be Unrelated Constructs

Many researchers and practitioners define procrastination as a conscious behavior (e.g., Pychyl and Flett, 2012; Chowdhury and Pychyl, 2018) that differs from unintentional or strategic forms of delay (e.g., Chu and Choi, 2005; Choi and Moran, 2009) in three key ways. First, a person must indicate their intention to procrastinate on a task. That is, the delay is goal-oriented rather than a means to an end. Second, this delay must be unnecessary or irrational. Third, the delay must result in negative consequences (Klingsieck, 2013). By this narrow definition, a student who delays studying to work on tomorrow’s homework assignment is not engaging in procrastination. Although the delay is intentional and may result in a negative outcome (i.e., poor test performance), it is a necessary consequence of wanting to perform better on the homework assignment. That isn’t to say this student hasn’t procrastinated at all—perhaps they delayed working on the homework assignment, despite their knowledge of the long-term consequences regarding their ability to study for their exam. Rather, this example illustrates an important point: only some delay is classified as procrastination.

Framed differently, procrastination represents a behavioral subset of the deadline action pacing style, which is characterized by increases in task engagement prior to a deadline. Given the relationship between the two constructs, researchers expected there to be a positive correlation between a person’s self-reported procrastination and engagement in the deadline action pacing style (Gevers et al., 2015). That is, everyone who self-reports procrastination should exhibit a deadline action pacing style, whereas only some people who exhibit deadline action pacing should endorse procrastination. This relationship was not observed in our study. One possible explanation is that pacing style and procrastination are not related constructs. This seems unlikely, given that delay is a defining feature of both procrastination and deadline action pacing. An alternative perspective is that one or both constructs are poorly defined.

Although a debate regarding the conceptual definitions of either construct is beyond the scope of this psychometrics paper, we acknowledge that this explanation would partially fit the pattern of results observed here. We observed that participants who endorsed statements relating to procrastination exhibited characteristic delays in research study completion but not in their overall patterns of research credit completion. Therefore, we recommend that future research address the competing hypotheses outlined here and give clarity to the conceptual definitions of procrastination and pacing styles.

### Some Self-Report Measures May Not Adequately Capture Procrastination

Another potential explanation for why self-report measures did not always predict behavior may have to do with the measures themselves. For instance, the results of our confirmatory factor analyses (CFA) and measure invariance (MI) tests indicated that certain scales (e.g., UPS IPS, PPS, TPS) displayed much better psychometric properties than other scales (e.g., MBP, AIP, APS). Consequently, it is possible that certain scales simply do a poor job of assessing procrastination, suffer from various validity issues (e.g., poorly written items, items that are unrelated to the underlying construct, etc.), and, therefore, are unable to predict meaningful outcomes (e.g., behavioral delay). Indeed, even the most well-validated personality scales can be laden with validity issues (Hussey and Hughes, 2020). Thus, it is crucial for researchers to ensure they are using appropriate measures when assessing procrastination. Only when measurement issues are eliminated as a potential explanation for the low self-report/behavioral delay linkage can more substantive explanations be properly evaluated.

### Limitations and Specificity of Findings

Finally, it is important to note that our findings are not without their limitations. We assessed procrastination in a specific context—research participation on a college campus—and it is possible that this is a domain for which some of the self-report instruments we elected to study are ill-designed. We also assessed procrastination at a single institution of higher education. While it is fair to say that our data indicate that respondents behaved similarly to those we have assessed at other institutions, it is possible that these findings may not generalize to other areas of the country or the world.

We also conducted our assessments on a subset of the many self-report and derivative instruments that are available to researchers today. While it is unlikely for single-item or subset items to perform better than a complete survey instrument (Ock, 2020), some researchers do use them. Additionally, some researchers recommend using refined, short-form instruments that assess a single dimension of the latent construct (e.g., the PPS, Svartdal and Steel, 2017; the GPS, Klein et al., 2019). We did not include these *ad hoc* or short-form assessments in our study. Given the evidence presented in this paper, we recommend that future research include such measures.

It is also worth noting that this study took place during the start of the COVID-19 pandemic. Although our behavioral measure of students’ pacing styles yielded results similar to those observed before the pandemic (Vangsness and Young, 2020; Voss and Vangsness, 2020), the overall proportion of students engaging in deadline action pacing was slightly higher (24% in this sample vs. 19% in Vangsness and Young, 2020). Future research might consider how external events impact students’ pacing styles and their self-reports of procrastination.

## Conclusion

Most of the instruments we included in our assessment were validated using tests of Cronbach’s alpha, exploratory factor analysis, and CFA. However, few instruments underwent tests of MI or predictive validity prior to their widespread use. The APA notes that “evidence of internal structure provides empirical support for the construct… it does not in and of itself establish [predictive validity], which requires additional evidence” tying scores to behavioral outcomes (Sackett et al., 2018). In our study, certain self-report instruments failed to meet these criteria (i.e., GPS, APS, PPS, AFP, AIP, UPS). Given these results—and those observed elsewhere (e.g., PASS, Yockey and Kralowec, 2015; APS, Pinxten et al., 2019)—we recommend that researchers cautiously employ these scales, especially when the goal is to predict behavioral delay within academic contexts. This may simply entail ensuring the scales have acceptable psychometric properties in the research context in which they are used and being transparent about any instances where appropriate psychometric properties are not obtained.

We do believe these results can be informative for future studies on the construct validity of these scales and for better understanding why certain procrastination measures display more/fewer construct validity issues than others. We also found that self-report measures were not predictive of students’ pacing styles. While it is possible that task completion strategies are not a viable measure of procrastination behavior, it is also possible that memory biases undermine participants’ reports of procrastination. Moving forward, we also recommend that future research be conducted to determine which of these hypotheses is correct.

## Data Availability Statement

The datasets presented in this study can be found in online repositories. The names of the repository/repositories and accession number(s) can be found below: https://osf.io/r7wcx/.

## Ethics Statement

The studies involving human participants were reviewed and approved by the Wichita State University IRB. The participants provided their written informed consent to participate in this study.

## Author Contributions

LV and NV jointly conceptualized the research idea. LV conducted and oversaw the investigation, conducted inter-item reliability, correlational, cluster, and predictive analyses, and was the curator of the OSF database where the study’s data files and supplementary materials are housed. VD and EM developed the study materials under the supervision of LV. NV conducted MI and CFA tests. LV, NV, and NM conducted the subsequent review and editing. All authors jointly prepared the original draft.

## Conflict of Interest

NV was employed by FMP Consulting. The remaining authors declare that the research was conducted in the absence of any commercial or financial relationships that could be construed as a potential conflict of interest.

## Publisher’s Note

All claims expressed in this article are solely those of the authors and do not necessarily represent those of their affiliated organizations, or those of the publisher, the editors and the reviewers. Any product that may be evaluated in this article, or claim that may be made by its manufacturer, is not guaranteed or endorsed by the publisher.

## Footnotes

1 Although other metrics (e.g., McDonald’s coefficient) are recommended for instruments with a high number of items, we used Cronbach’s alpha to facilitate comparisons with published literature, which uses this measure of internal reliability almost exclusively.
